# End-of-Life Care of a Patient With Pontine Stroke and Prolonged Hospitalization: A Case Report

**DOI:** 10.7759/cureus.59351

**Published:** 2024-04-30

**Authors:** Stephen Howard, Ryan Tam, Mohammad Naser, Mohammad Jawish

**Affiliations:** 1 Physical Medicine and Rehabilitation, Lake Erie College of Osteopathic Medicine, Elmira, USA; 2 Physical Medicine and Rehabilitation, Rochester Regional Health, Rochester, USA; 3 Internal Medicine, Lake Erie College of Osteopathic Medicine, Elmira, USA; 4 Internal Medicine, Rochester Regional Health, Rochester, USA

**Keywords:** code status, medical ethics, end-of-life decision-making, prolonged length of hospital stay, stroke

## Abstract

Stroke is a leading cause of long-term disability in the United States that can lead to loss of function and consciousness. With the abrupt onset of the brain insult, end-of-life care discussions are an important attribute of respecting the patient’s best wishes and upholding the ethical principles of autonomy, beneficence, nonmaleficence, fidelity, and justice. Furthermore, the topic of extending life support to individuals with poor prognostic factors of improvement in quality of life and functional recovery has been a continued topic of debate due to a multitude of factors, including the wishes of the patient, familial emotions, cultural beliefs, and religious influences. This case involves a patient who suffered from a left pontine stroke, necessitating multiple end-of-life care conversations. Despite no clinical improvement for several years, the patient required prolonged hospitalization and ongoing ventilator use.

## Introduction

One of the leading causes of death and long-term disability for patients in the United States is stroke, including acute ischemic stroke and intracerebral hemorrhage [1]. Thus, end-of-life care discussion, including code status, chest compressions, and ventilator use, is important for patients who experience stroke. Healthcare providers are often confronted with matters of contention in managing end-of-life care for patients with strokes regarding discussing their goals of care with a surrogate decision-maker [1]. Additionally, it has been shown that in the United States, approximately 8.8% of patients who experience acute ischemic stroke require percutaneous endoscopic gastrostomy (PEG), with over 50% of these PEG placements occurring within the first week of admission. Approximately 10% to 15% require mechanical ventilation with a tracheostomy, which is often performed within the second week following the onset of a stroke [1,2]. Consequently, a discussion regarding the need for nutritional and airway management has become necessary for some patients who experience strokes.

Pontine infarcts are oftentimes associated with high rates of morbidity and mortality, with varied clinical presentations depending on the afflicted region, whether it be ventral caudal, mid-pontine base, or tegmental. This accounts for approximately 15% of acute vertebrobasilar ischemic strokes and 7% of ischemic infarctions [3]. With the specific location of the infarction, the symptomatic presentation can consist of pure motor hemiparesis/hemiplegia, pure sensory stroke, respiratory and cardiac dysfunction, decreased consciousness, and/or locked-in syndrome [4]. Given the substantial impact that strokes can have on an individual’s quality of life that can cause great suffering and grief to both the patient and their family member, healthcare providers must display empathy and uphold the ethical principles of autonomy, beneficence, nonmaleficence, fidelity, and justice [5]. This further holds true for severe cases of acute brain insults, as it typically first manifests with a coma that can then undergo transformation to a condition of persistent vegetative state characterized by the inability to make contact or express awareness [6].

In the context of this case report, we present a patient with a complex medical history who is hospitalized for a prolonged period of time in a persistent vegetative state on prolonged ventilator support after sustaining a pontine infarction with a poor prognosis for recovery.

## Case presentation

**Figure 1:**
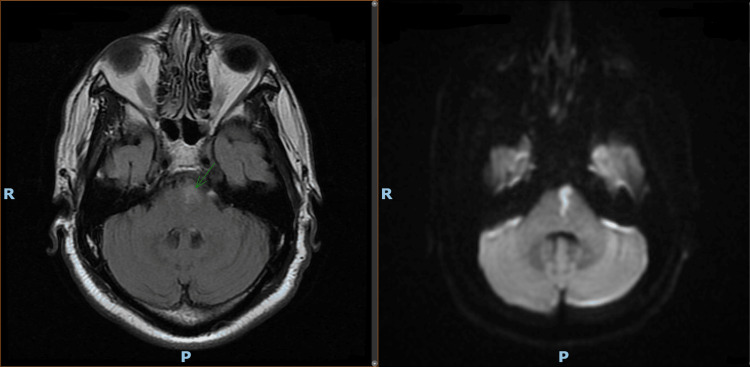
Axial T2 FLAIR MRI (left) and diffusion-weighted imaging (right) There is a small area of acute/subacute infarct in the left paramedian pons seen on T2 FLAIR MRI (green arrow). Diffusion-weighted imaging shows the same infarct.

This is the case of a 64-year-old male with a past medical history of multiple sclerosis, diabetes mellitus, hypertension, hyperlipidemia, chronic obstructive pulmonary disease, chronic kidney disease on hemodialysis, and nicotine use who presented to the emergency room with an acute-onset right-sided weakness 30 minutes prior to arrival. He was sitting on his porch acting “normal” when his sister was with him and noticed sudden vomiting, an inability to speak, and right-sided weakness. Prior to this presentation, the patient was able to function independently. The patient's vital signs were stable on arrival. On physical examination, the patient was aphasic, with upper and lower extremity right-sided weakness. The patient was stabilized and admitted to the hospital for further evaluation. Initially, computed tomography of the head without intravenous contrast was performed, and a non-acute insult with nonspecific small vessel ischemic changes was demonstrated. This required further attention from the neurology stroke alert team, which recommended the administration of a tissue plasminogen activator. He had an axial T2 fluid-attenuated inversion recovery magnetic resonance imaging (FLAIR MRI) that later demonstrated infarction at the left paramedian pons, as shown in Figure 1. The findings of this acute infarction were compared with diffusion-weighted imaging.

Following this hospitalization, he then had multiple subsequent hospitalizations for respiratory, neurological, and other complaints, such as progressive dysphagia, requiring a percutaneous endoscopic gastrostomy tube insertion. Subsequently, he was admitted to the hospital with worsening recurrent encephalopathy secondary to multiple infections and progressive infected wounds, resulting in declining cognitive function. This patient was hospitalized for more than 600 days with worsening cognitive function and progressed to a comatose state. The hospital course was complicated by recurrent infections and gastrointestinal bleeds.

He was then transferred to another hospital, with the current length of stay being greater than 500 days. The patient is currently in a persistent vegetative state with a poor prognosis. His prolonged course has been complicated by recurrent pneumonia and recurrent pyrexia. He is being treated for ventilator-dependent chronic respiratory failure, severe peripheral artery disease, multiple sacral and buttock ulcers, foot gangrene, and pyocystitis. His mechanical ventilation via tracheostomy requires continuous respiratory therapy and a pulmonologist for patient follow-up. He has developed multiple pneumonias, required multiple courses of antibiotics, and developed multi-drug-resistant organisms. 

The patient's code status remains full code, and his family, despite numerous discussions and counseling, has declined to sign the Medical Orders for Life-Sustaining Treatment (MOLST) form to change the patient's code status from full code to do not resuscitate (DNR) and do not intubate (DNI). Multiple family meetings occurred between treating clinicians and their families, discussing the goals of care and the poor prognosis and outcome. The patient is still receiving inpatient care with a plan to be transferred to a long-term facility, which has been a big challenge given the need for mechanical ventilation and hemodialysis.

## Discussion

As in the case presented above, patients who experience pontine infarcts may exhibit years of complications following the inciting event. Patients in a persistent vegetative state, characterized by eye-open unconsciousness and sleep-wake cycles, are unable to have awareness of themselves or their surroundings. Meaningful recovery of consciousness and good function is highly unlikely after three months, and the average life expectancy is two to five years, given the preservation of hypothalamic and brainstem functionality [7]. In the case where medical futility is evident, an end-of-life discussion should be initiated with the patient's healthcare proxy.

This patient is currently undergoing years of hospitalization with multiple recurrent infections, which raises the significance of goals-of-care discussions with the family and surrogate decision-maker. Physicians have a crucial role in delivering clear communication to facilitate the end-of-life care discussion. With an interdisciplinary team approach, the patient can be offered realistic options when medical futility is evident [8].

It has been shown that medical healthcare providers are poor at predicting a patient’s quality of life following a stroke, particularly due to limitations in prognostic uncertainty, communicating goals of care, and advanced care planning documentation [1]. An understanding of this matter of contention can help physicians guide patients and their families to optimize care. It has been recommended that clinicians ensure that the relevant people are involved in meetings when discussing the goals of care, asking the surrogate decision-maker how they would like to use their role, and using an “ask-tell-ask” approach to ensure understanding [1]. Proper communication is essential in this process, especially with patients who have experienced strokes, given the uncertainty of the long-term prognosis. Acknowledging this uncertainty by describing the “best and worst possible scenario” with the surrogate decision maker and evaluating any advanced care directives or verbal wishes of the patient can better optimize patient care [1]. In the case described above, due to his prolonged hospital stay, hundreds of medical providers were involved in his care, which makes maintaining consistency in communication with the patient’s surrogate decision-maker difficult. This may have led to confusion with the family in understanding ethical considerations. 

This case brings an important discussion on how physicians should approach the end-of-life care discussion with family members in patients with poor prognosis and those requiring long-term mechanical ventilation support. It has been shown that family members consider the main responsibility of the decision for DNR orders to be from the physician and that physicians may not pay enough attention to the role of the patient’s family in this respect [9]. In this case, the patient’s family wished for life-prolonging treatment for a patient with a severely compromised medical condition. As a result, this patient has been admitted to many different hospitals, with a length of stay of over 1100 days. Physicians are able to withhold cardiopulmonary resuscitation against family wishes in Canada, according to the “Joint Statement on Resuscitative Interventions'' [7]. This raises an important question, is it ethical for physicians to supersede the wishes of the family on the grounds of futility?

## Conclusions

Physicians play a pivotal role in elucidating the clinical status of hospitalized patients and helping facilitate end-of-life discussions when the need arises. The decision to prolong life-sustaining treatment or pursue comfort care for patients with comorbid conditions with poor prognostic value should be based on a patient-centered approach that takes into account their best interests. Yet, this is a controversial topic of discussion as there are no clear-cut standardized guidelines thus far in the United States that are adopted nationally to help dictate care management. Hence, we hope this case report provokes discussion within medical providers on how to approach a patient when medical treatment is futile and the designated healthcare proxy is faced with the dilemma of making the best-informed decision. Additionally, it is important for physicians to not only display empathy in these difficult decisions but also to champion guidelines that other countries have already pre-established.
